# Stakeholders understanding of the concept of benefit sharing in health research in Kenya: a qualitative study

**DOI:** 10.1186/1472-6939-12-20

**Published:** 2011-10-03

**Authors:** Geoffrey M Lairumbi, Michael Parker, Raymond Fitzpatrick, English C Mike

**Affiliations:** 1 Kemri-wellcome Trust Research Programme, Centre for Geographic Medicine, Nairobi Unit, P.O Box 43640, 00100, Nairobi, Kenya; 2 Ethox Centre, University of Oxford, Oxford, UK; 3 Department of Public Health, University of Oxford, Oxford, UK

## Abstract

**Background:**

The concept of benefit sharing to enhance the social value of global health research in resource poor settings is now a key strategy for addressing moral issues of relevance to individuals, communities and host countries in resource poor settings when they participate in international collaborative health research.

The influence of benefit sharing framework on the conduct of collaborative health research is for instance evidenced by the number of publications and research ethics guidelines that require prior engagement between stakeholders to determine the social value of research to the host communities. While such efforts as the production of international guidance on how to promote the social value of research through such strategies as benefit sharing have been made, the extent to which these ideas and guidelines have been absorbed by those engaged in global health research especially in resource poor settings remains unclear. We examine this awareness among stakeholders involved in health related research in Kenya.

**Methods:**

We conducted in-depth interviews with key informants drawn from within the broader health research system in Kenya including researchers from the mainstream health research institutions, networks and universities, teaching hospitals, policy makers, institutional review boards, civil society organisations and community representative groups.

**Results:**

Our study suggests that although people have a sense of justice and the moral aspects of research, this was not articulated in terms used in the literature and the guidelines on the ethics of global health research.

**Conclusion:**

This study demonstrates that while in theory several efforts can be made to address the moral issues of concern to research participants and their communities in resource poor settings, quick fixes such as benefit sharing are not going to be straightforward. We suggest a need to pay closer attention to the processes through which ethical principles are enacted in practice and distil lessons on how best to involve individuals and communities in promoting ethical conduct of global health research in resource poor settings.

## Background

The concept of benefit sharing in health related research, which at a minimum, entails the elucidation of what if anything, is owed to participants, their communities and host nations that take part in such research, has recently emerged as an important strategy for addressing moral issues of relevance to individuals, communities and host countries in resource poor settings when they participate in international collaborative health research [1-3]. This concept requires consideration of a new ethical issue, the social value of research [4], by involving research participants and the researched communities in ensuring that global health research undertaken in resource poor settings is not exploitative and is responsive to the expressed needs of such settings. Benefit sharing was developed as a strategy to forestall the potential for exploitation of individuals, groups and even countries that host research and provide such groups with reasonable returns, thus promoting the social value of the research [5].

Concerns over the exploitative potential of global health research arose from the realisation that: i) the amount of clinical and other health related research being undertaken in developing countries had increased, in itself a positive move, arising from advocacy associated with the 10/90 gap [6,7], ii) that advances in science and technology especially the computing and statistical capabilities that made Genome Wide Association (GWAs) studies possible not only to implicate individuals, but also whole populations in research [8,9] and, iii) the understanding that the pathway between knowledge generation and improved care is not straight forward [10-12].

The potential of the above developments to produce unfairness is exacerbated by the fact that most resource poor settings are also characterised by high unmet healthcare needs, weak capacity among ethical and other research regulatory systems to safeguard the interests of research participants, and an often poor exchange model capable of translating research findings into improved health [4,13-15]. The argument has been that although these conditions are not sufficient for research to be exploitative, they nonetheless predispose participants in resource poor settings to exploitation.

The consideration of the social value of research and the need to engage with benefit- sharing within health related research as strategies to address the potential for exploitation have important similarities. The two call for prior consideration of the moral obligations of researchers (and funding institutions) that arise once individuals and groups participate in health related research, including the identification of the benefits to be shared, the beneficiaries and where the responsibility for providing the benefits lie. These strategies have influenced the conduct of collaborative health research as evidenced by the number of publications and research ethics guidelines that require prior engagement between stakeholders to determine the social value of research to the host communities [14,16-19]).

While efforts have therefore been made to produce international guidance on how to promote the social value of research through such strategies as benefit sharing, the extent to which these ideas and guidelines have been absorbed by those engaged in global health research especially in resource poor settings remains unclear. The work reported here aimed to explore awareness of the concept of benefit sharing among stakeholders involved in health related research in Kenya.

## Methods

This study was conducted among stakeholders drawn from within the broader health research system in Kenya. Overall, the Kenya Medical Research Institute (KEMRI) is the main body responsible for health related research in Kenya through its ten (10) research centres located in different parts of the country. KEMRI is mandated to carry out research into human health and generate evidence to improve health status in Kenya and globally. The Institute mainly undertakes research in areas such as infectious diseases; parasitic diseases; epidemiology, public health and health systems research; and biotechnology and non-communicable diseases. Besides KEMRI, several other bodies actively conduct health related research in Kenya, key among them, private and public hospitals, and universities with Health/Medical Sciences departments. Hospitals that undertake health related research include; Kenyatta National Hospital (KNH) in conjunction with the College of Health Sciences of the University of Nairobi, and Moi Teaching and Referral Hospital (MTRH) in conjunction with Moi University. In addition, there are local networks, international research institutions, pharmaceutical companies, charities/foundations and International Non Governmental Organisations (NGOs) that equally conduct research related to healthcare. The accounts that we report here were articulated by researchers, members of Institutional Review Boards (IRBs), policy makers, civil society organisations, research sponsors and community representative groups within such institutions or impacted by their research. We have previously reviewed the recommendations made by various international and region specific research ethics guidelines regarding benefit sharing [20] as a framework of theory that might be expected to guide how the research system acts in real settings. In the review, we argued that there is less consensuses among research ethics guidelines, regarding the specific responsibilities of researchers over what is ethical in promoting the social value of research. We further noted that this was likely to have practical implications for efforts aimed at enhancing the social value of global health research undertaken in resource poor settings.

### Selection of key informants and data collection

Key informant and in depth interviews were conducted with 52 respondents drawn from institutions described above. Respondents were grouped into 6 categories (see table 1), representing actors that are expected to continually engage with ethical issues arising from the conduct of health related research. These groups might be expected to be involved in negotiations relating to the promotion of the social value of research, and the consideration of research benefits in particular.

**Table 1:** Categories of participants and number of interviews under each

Participating institutions	Number of Interviews
Research institutes & University depts. with health science departments/schools/institutes	16
NGOs with research functions	3
Policy makers	4
Ethical review bodies & Research coordinating bodies	11
Pharmaceutical Firms & Other funding bodies	7
Civil Society Organisations (CSOs) &KEMRI community representative/Advisory groups	11

We did not pre-specify formally the number of interviews that would be conducted. Instead we adopted an initially selective approach where respondents that were deemed likely to have engaged with issues relating to benefit sharing were theoretically sampled as the study proceeded [21]. Thus the final number of interviews from the different categories was determined by the standard principle in qualitative research of data saturation, the point at which no new ideas, views or experiences were being expressed.

Data collection was undertaken over a six month period between October 2007 and April 2008. Potential respondents were contacted by telephone, to discuss the study objectives and inclusion criteria. After accepting this preliminary invitation formal participation in the study was confirmed at the time of interview when each respondent was asked to provide individual, written informed consent. Interviews were conducted face-to-face and conversation recorded together with detailed notes of the discussion. The interviews were conducted by the principal investigator (LM), except for those conducted among members of a community representative groups which were conducted with the help a research assistant who was more conversant with Kiswahili. The assistant was trained on how to conduct qualitative interviews and was accompanied by the principal researcher during these interviews. Interview guides were prepared based on literature review, analysis of research ethics guidelines and initial findings from the pilot phase.

The purpose of the research was explained as an exercise to understand how the importance of research/study was communicated to research participants or framed including why it was important to participate in research. Respondents were therefore asked to reflect based on research they were involved in, over what they considered to be the benefits, the beneficiaries and the obligation of various actors to provide the benefit. Participants who did not have an immediate experience to draw from were invited to reflect based on two examples of clinical trials; Malaria and HIV/AIDS clinical vaccine trials. The interview guides were therefore framed around stakeholder's views of the benefits, beneficiaries, obligations and how such decisions were made. All respondents except community representatives were however asked whether they had come across the term "benefit sharing", as a way of teasing out their level of engagement with the debate over fairness in global health research. The interview process was the main tool for interviews exploring how stakeholders understood the new concept of benefit sharing within global health research. Initial observations from the pilot, revealed that respondents were often unfamiliar with the language and issues relating to benefit- sharing, requiring initial conversations to explore and develop the topic idea.

The interview encounter was therefore the ultimate context within which to ensure production of valid knowledge (the emphasis was not on observation, but rather, conversation and interaction). This was made possible by interviewing reliable "witnesses" who were actively involved in health related research and the use of valid documents (such as the research ethics guidelines) and arguments as part of the social interaction.

### Data management and analysis

Audio recorded interviews were transcribed verbatim and translated where necessary. Analysis was done following a grounded theory approach [22]. Preliminary analysis entailed progressive coding and categorisation of issues emerging from the interviews. The transcripts were read several times in order to identify meaning units for developing an organising system for coding the data. We developed our organising system following a criterion suggested by Srivastava and Hopwood, [23], which involves exploring data alongside the issues people were talking about in the interviews, the initial objectives of the research and the contradictions between the two. The analysis proceeded from open coding, comparing transcripts and coding into each other to the point of saturation and later organising the codes into themes.

### Ethical Considerations

Ethical approval for this work was granted by the national ethics review committee in Kenya (NERC). Informed consent was obtained from all respondents, including the permission to record the conversation. In most cases, respondents were happy with the recording, except for a few who declined. Other ethical considerations were the need to protect the confidentiality of the respondents, which were potentially revealing because of the small sample, and the nature of the people interviewed. In this case, all the interviews were anonymised by assigning pseudonyms and any attribution was made to the category from which the respondents were recruited. In addition, ethical issues arising from data representation and interpretation were considered, and every attempt was made to stay close to the data without distorting the salient differences between and among the categories of respondents.

## Results

We present below, the stakeholders' awareness of debates within academic and regulatory literature relating to benefit sharing, as well as the moral issues that are seen to arise from the conduct of collaborative health related research in resource poor settings. We later examine the extent to which these views have anything in common with those being discussed in the bioethics and regulatory literature about benefit sharing while pointing to some of the implications for this understanding.

### Awareness of debates relating to benefit-sharing

In most cases interviews started with questions meant to explore interviewees' awareness of academic and regulatory debate about the fair sharing of benefits in international research undertaken in developing countries. Stakeholders were asked whether they had come across the term benefit sharing in the context of research ethics, primarily, to tease out their familiarity with the debates leading to the emergence of the need to promote the social value and benefit sharing as ethical imperatives. In this particular instance the term 'social value' is used in a broad sense to include all the questions about the value/benefits of the research to communities including its relevance to their health care needs. Seen from this perspective, benefit sharing is one possible way of making research to have social value.

### At the research level: Awareness among researchers

*"...Benefit sharing? No, I know the English word but I do not know the exact terms... "(Researcher 2)*.

The quote by Researcher 2 captures a typical response commonly given by researchers involved when asked whether they had come across the concept of benefit sharing.

On several occasions our respondents seemed unaware of the meaning of the term 'benefit sharing' although they insisted that they could infer the meaning from its etymological roots as opposed to its usage in ethics literature. In other cases, some researchers reported that they had not come across the term 'benefit sharing' as illustrated by the following excerpts of the discussion between Researcher 7 and the interviewer.

Interviewer: Have you come across the term benefit sharing before?

*Response: Benefit sharing? No*.

Interview: What do you think it is?

Response: That's a hard ...., Benefit sharing... I guess it's both the participants benefitting from the study, as the study benefits the clients, and the community, probably... (Researcher 7)

Apart from the outright lack of awareness of the ethical concept, some researchers reported that they never bothered with the finer details over issues relating to benefit sharing but instead, operated on the assumption that their institutions had taken necessary steps to operationalise it. The following quote by Researcher 15, while indirectly illustrating some sense of awareness, portrays a rather distanced engagement with the practical considerations relating to benefit sharing.

...*what I know is that at the institute level, they are coming up with the Intellectual Property Rights and Benefit Sharing between the institute and the inventors. This will take care of such issues and we don't have to worry....so the institute will also play its role as a place to which we were affiliated and it will also have its benefits (Researcher- 15)*.

In yet another instance, a researcher noted that there was some unspoken assumption among researchers that since research protocols are approved by respective institutional review boards including Research Ethics Committees (RECs) the issues concerning benefit sharing had already been addressed, as illustrated by the following quote;

... *Ok, since the research that we do, has to be approved by the ethical review committee essentially both the scientific committee and ethical review committee where we usually have to explain the sort of benefits they are likely to get directly in the short term and also you need to explain to them even the long term, how they stand to benefit.... but most of the times we leave it there... (Researcher3)*.

As expected, there were researchers, although clearly a minority, who reported some awareness of the concept as articulated in the quote by Researcher 13.

*"...of course we have considered issues related to what benefits come out of this research because it is an issue that volunteers have been raising. However this has not been explicitly addressed as has been other issues. When we started these vaccine trials here, there was no framework in the country to guide our operations..." (Researcher13)*.

Research regulatory level: Awareness among members of the IRBs

...*the question of benefit sharing particularly in the third world is a very tricky one. From a general point of view the complaint has generally been that in collaborative research in particular, the relationships say between those in the third world and those in the first world has always been tilted in favour of the first world and that therefore works a lot of injustice. ...this makes pursuing benefit sharing quite complex although in most of the International forums where this has been discussed it is clearly there*... (Institutional Review Board 1).

Members of the Institutional review boards unlike researchers showed some level of awareness of the concept, although they were perplexed over the practical implications over its consideration in research conducted in Kenya. The above quote was a response to how consideration for benefit sharing was handled within protocols submitted by researchers. Similar challenges relating to lack of clarity over practicalities of engaging with benefit sharing were further articulated by members of different IRBs, who while responding to a similar question observed that;.... *It is very tricky. And, I have been engaged in this debate (on benefit sharing) myself and I have said yes, it is good but, let's be practical. Practically the implementation becomes impossible... (*Institutional Review Board 2). While these quotes by members of IRBs illustrate concerns over feasibility of engaging with benefit sharing in resource poor settings they nonetheless show some degree of familiarity with the concept. Although this degree of awareness is expected among members of IRBs, their divergence in opinion on the practicality of engaging with benefit sharing in resource poor settings was striking, especially, considering that both IRB 1 and IRB 2 were drawn from the two main bodies with a final mandate for ethical review of research protocols in Kenya.

### Awareness at the community level: Awareness among CSOs and other Community Representative Groups

...*Benefit sharing is more or less taking ownership of what comes out let's say of an intervention or something like that, like what you are saying like if we have to have collective responsibility towards the fight against HIV and Aids and out of that you find the prevalence rates are going low, infections are quite reduced, there are few infections or there is better life for those who are infected...you see you also benefit, but we are also benefiting as a large collective entity who put in collectively *... (Civil Society Organisation 3).

Awareness of issues relating to benefit sharing at the community level was explored by interviewing respondents drawn from Civil Society Organisations (CSOs)s and other community representative groups including members of Community Advisory Boards (CABs) and the KEMRI Community Representative (KCR) group. The CSOs, CABs and the KCR were expected in theory to champion community interests in research.

Another respondent from a CSO that was involved in championing community interests within research aimed at developing interventions to address malaria, TB and HIV noted that;

....*I think benefit sharing refer to a situation where all the parties involved in a given situation feel proud for the success that has been achieved, they feel part and parcel of the mechanism and the system that has brought about that success. So they feel more or less like they have made life or the environment leaving it a better place than it was initially...It is taking ownership of the success*. (Civil Society Organisation 5).

As was the case with other stakeholder groups there were limited instances where respondents showed awareness of issues relating to benefit sharing, as illustrated by the quote by Civil Society Organisation 2;

...*Of course this is a subject, which has been recognized and debated on the whole issue of benefit sharing, while in the sense of clinical trials or in the sense of other research whether its agricultural research, or biodiversity research where you know at the end of the research there is some kind of commercial product, I think it depends on what is being looked at. In the area of clinical research, the potential benefit from such work, in most cases is pharmaceutical products, this could be a drug, it could be a biological product, and it could even be a medical device that is used in treatment, or could be a vaccine used in prevention, that's the product*... (Civil Society Organisation 2).

In theory, this awareness, albeit limited, demonstrate the extent to which some groups are able to discharge their mandate of representing community interests within global research enterprise. Broadly these responses clearly show that there is little formal exposure or joined up thinking with regard to the practical aspects of interpreting abstract ethical guidance on the ground.

Although respondents made only limited use of the concept of benefit sharing as it is emerging in formal ethical discourse, they nevertheless expressed a number of views and concerns about health research that might be considered to point towards the concept. We present exemplars of these views below.

### The scope for exploitation

*"... There is disease, there is plenty of population, and there is poverty..., there are plenty of poor people who know nothing and who do not know their rights. So that is why it is an easy field to come in and come out. Parachute in, Parachute out...they might be doing something else also without you knowing. If they are drawing blood samples, they're telling you we are doing it for Malaria, but do anybody have control over it, that those blood samples are not going out and they are doing some genetic testing or they are doing something else on it? /.../ that is my fear and concern..." (Institutional Review Board 5)*.

The quote is an acknowledgement that both individuals and communities participating in research were vulnerable and therefore in need of protection. Such explanations were given as the main reason for considering ethical issues in medical research. *"...Ideally there is need to ensure that researchers do not take advantage of the subjects and... (ensure that)... whatever they are doing is acceptable both ethically and scientifically" (Researcher 4)*.

The potential for exploitation was further articulated by reference to the common features that characterise resource poor settings, including the huge unmet healthcare needs, poverty and lack of a functional research regulatory system, the above quote by Researcher 4 captures some of the concerns among members of the IRBs that health related research might inadvertently promote unfairness among certain classes of people in such settings. The metaphor of a parachute appears to have been used here to illustrate the absence of long-term relationship with the researched community, either in understanding their needs (prior engagement) or any thought about the impacts of research. It is however important to note that there are situations in which long-term relationships between research institutes and communities have been established and this perhaps generates different obligations. The remarks above were made in reference to the concern over vulnerability, and the attendant need for protection against exploitation as previously noted. While these quotes broadly speaks about the need to protect communities from exploitation, it goes beyond the vulnerability produced by conditions of poverty, to also address the potential for local researchers to involve communities in research that is scientifically and ethically sound, but still lacking value to such communities. Where there are conditions of poverty, limited knowledge and the absence of strong mechanisms to govern and regulate research activities, consideration for benefit sharing is seen as being capable of establishing standards to avert crises that are likely to arise from participation in global health research by local communities.

### Addressing immediate health care and other societal needs

*"...I'd say that the people here come against economic difficulties, that if one involves himself in that research, he expects to be taken care of in terms of access to health/.../for instance, if a child is ill and needs to go to hospital they will get help from the research team..." *(Kemri Community Representative 5).

The second concern relates to the need to use resources associated with research in addressing the material conditions of communities that host/participate in research as illustrated by the above quote by a member of a community representative group.

Similar concerns were echoed by some locally based researchers who reported that while the provision of health care services is not the primary goal of research, the dichotomy between research and other social issues should not be stringently applied in poor settings where people have high unmet health care needs.

*"...I know it's still like the Governments' thing to do some of those things, but...you should not say I think this is for research, when another person is dying and you have the medicines, I think it's not fair. Even if you have the equipment for research, it can be used for diagnostics if it's so needed." *(Researcher 2).

From this appeal to expectations which are brought into the research process by research participants and the call to disregard the dichotomy between research and health care provision, it appears that some of these stakeholders expect the resources associated with research, to be used for responding to the needs of the community, including those related to health care.

### Future needs: the importance of access to future interventions and treatments

*"... we are learning and we have learnt something from previous research, we have to act now by looking ahead of the vaccine trials as opposed to waiting until the end of the trials and ask ourselves, what will happen in future and start asking those questions ourselves; how will Kenya benefit? How will those individuals benefit? If we can answer those questions now it will be so nice and save us a lot of problems" (*Civil Society Organisation *1)*.

Apart from responding to immediate health care needs, some respondents saw opportunities from participation in health research as potentially securing future access to proven interventions once research was complete. The emphasis on learning and the time dimension for planning were made in reference to HIV/AIDs vaccine trials that were previously undertaken in Kenya but later culminated in disputes over ownership of the results and the expected products pitting the collaborating institutions on one hand, and the research participants on the other.

*"... It was not an easy fight to get the pharmaceutical companies to see this point of view to recognize the global corporate social responsibility, to sensitize them to the need and of course there are a lot of people who are suffering from these diseases in the developing world, who are unable to access these drugs because they cannot afford it..." *(Civil Society Organisation 2).

The importance of forward thinking as a way of promoting future access of research benefits was equally underscored by reflecting on previous struggles between civil society organisations and the pharmaceutical industries. By making reference to previous drug trials conducted in resource poor settings, which led to the development of effective antiretroviral therapies (ARTs), the quote by Civil Society Organisation 2 demonstrates the potential of benefit sharing to address future community needs relating to proven interventions.

### Research and sharing profits

*"...I think its business, in my view. Lately I've come to understand that research especially big time research out there is a huge industry. And people will put money on what they see as potentially giving back to them in the future..." *(*Institutional Review Board *3).

Other respondents regarded consideration for benefit sharing as a tool to foster recognition of the huge opportunities for investment presented by research and the need to enjoin all stakeholders in reaping the benefits. The quote by *Institutional Review Board *3 above for instance, represents a concern expressed by local level stakeholders (including members of community advisory boards, community representative groups, researchers and the IRBs) to liken health related research to a business venture whose goal is the pursuit of profit.

The stakeholder accounts presented above therefore underscore the importance of fairness especially in the context of recognising contribution made by research participants and their communities, by participating in the development of medical and public health tools through research.

### Developing community support

*"... What is the benefit of this for us? You will realise that for the last almost 12 years and especially in the previous decade (the industry) was seen to just purely profit from human suffering and people had a very negative view of the Pharmaceutical Industry. And in the last 10 years, we are sort of re-modelling our self and trying to make sure we project our self in a very responsible manner*..." (Pharmaceutical company1)

On the other hand, some stakeholders especially those associated with the funding of health research appealed to the importance of cultivating community support and goodwill by responding to the previous negative press that characterised the conduct of collaborative research in resource poor settings as aptly summarised by the above quote by Pharmaceutical company1.

### Resource and risk pooling

*"... So our R&D people are coming from the Industry, so we understand it. The multinational big Pharmaceuticals, if they want to do something there because they see the need, they also see the urge from the Global Community, they want to limit their financial exposure in the R&D at least part of the cost are borne by someone else, by the Public Community and also far as the risk, if one Project fails then maybe there will be other Projects coming afterwards" *(Public Private Partnership 2).

A related benefit for considering benefit sharing was the need to develop a collective approach to enlisting community support by pooling together resources that can be invested in the development of medical and public health research whose return on investment (ROI) was not guaranteed as summarised by Public Private Partnership 2 above. This particular quote a voice of a stakeholder from a public private partnership, commenting on how appeals to collective resource pooling between the public and private enterprises had successfully been used to address challenges associated with the funding of research into "neglected diseases". Prior to the establishment of partnerships between the public and private ventures, pharmaceutical industries mainly relied on a pure business logic/model in making decisions to invest in the development of health interventions. Such investment decisions were the subject of debate and criticisms, following the publication of the 10/90 report (the statistical finding by the Global Forum for Health Research, that only ten per cent of the world expenditure on health research and development was devoted to the health problems that primarily affect the poorest 90 per cent of the world's population) on Health Research by the Global Forum for Health Research. In response, the pharmaceutical industry teamed with other players to develop alternative funding mechanisms to facilitate research into diseases of public health importance to resource poor settings.

### Making actors accountable/socially responsible

In addition, there were concerns touching on accountability among actors involved in research as illustrated by the quote by *Institutional Review Board *5.

*"... I think you should be realistic, that nobody is going to come back and you may not be there to enforce that somebody should give back to the community...and once they find what they are looking for, they may just take off..." *(*Institutional Review Board *5).

The concern over whether research sponsors are likely to be accountable to the host community especially in the absence of strong governance and regulatory mechanisms was a key consideration among IRBs and other local level stakeholders. More importantly, the quote underscores the underlying fear that some actors might take advantage of weaknesses within these regulatory mechanisms, and exploit local communities by failing to make research benefits available.

## Discussion

In this study we aimed first; to explore the understanding of the concept of benefit sharing among stakeholders involved in health related research in Kenya and second, to examine the extent to which this understanding had resulted in practical steps to protect the interests of individuals and communities who participate in health related research in Kenya. In order to achieve this, interviews were conducted with stakeholders that often influence the ethical conduct of health research in the country.

One of the anticipated outcomes was that stakeholders would show awareness [24]of the debates relating to benefit sharing and the promotion of the social value of research, and the internal logic for engaging with benefit sharing within global health research. What our findings however show is that in most cases, there is a very limited formal engagement with recent mainstream thinking over the need to promote justice and equity in global health research.

Clearly, there has been significant debate at the international level over the last decade, regarding the ethics of global health, including promulgation of various research ethics guidelines [25]. Despite this, it appears that most stakeholders key among them researchers, IRBs, policy makers, regulatory bodies and CSOs have largely been passive recipients of these ethical recommendations. The apparent disconnect between researchers and IRBs is for instance quite revealing, because IRBs are in theory expected to provide guidance to researchers in operationalising ethical requirements in practice. Both researchers and IRBs are expected to play a key role in promoting ethical conduct of health related research involving human subjects.

Our findings further suggest that despite this limited engagement with the formal thinking within global health research, stakeholders have a number of key moral concerns associated with participation of poor communities in international research. These concerns included a sense of the need to ensure fairness in the distribution of benefits arising from research. Demands for fairness might be attributed to several other factors, including some that are political in nature, but there was also a growing sense in which health related research was regarded as a joint activity not only involving researchers and sponsors, but also other players including the host communities. Such perceptions seemed to be based on a belief that research involves the active participation of communities and participants as something like 'co-producers', where the actors involved in whatever capacity are entitled to a stake in the proceeds derived from research. The metaphor of co-producing is important, in the sense that facilitating research either by giving information or body tissue is in itself seen as a form of capital, and as is the case in any act of production, contribution must be rewarded accordingly, which potentially pushes participation in research away from volunteerism. Interestingly, claims over co-production are not confined to types of research that are aimed at producing commercial products, but were also common in other types of research such as epidemiology and health systems research. Similar notions of members of the collective as co-producers have been described elsewhere [5,26]. Promoting fairness in the distribution of proceeds from research has for instance played a critical role in pushing for recognition of the interests of different actors involved in research and bringing them to the negotiating table [5].

Other concerns that were voiced relate to demands for some of resources tied to health research, and particularly international collaborative research, to be channelled to addressing unmet health care needs of the local community [3,27,28], and the need to protect poor communities against exploitation. Lastly, by talking about the importance of putting in place strong and binding mechanisms that ensure that agreements made during the negotiation are honoured, these actors were perhaps pointing to the lack of accountability and trust in the manner in which research had previously been conducted.

Whilst respondents made many observations indicating the importance of benefits to individuals and communities of those participating in health research, their comments also showed that practical implementation of benefit sharing would be complex, contentious and difficult to achieve.

These findings have several practical implications for global health research in general, and engaging communities in research, especially in the context of collaborative health related research in resource poor settings. Firstly, they demonstrate the fact that while in theory several efforts can be made to address the moral issues of concern to research participants and their communities in resource poor settings, careful thought should be given on the practical aspects of implementing strategies such as benefit sharing. Secondly, the findings point to an urgent need to pay particular attention to the processes through which communities and other stakeholders are recruited into biomedical research as a starting point towards developing better strategies of promoting participation and engagement in global health research. Thirdly, they demonstrate that while rapid theoretical leaps can be made in promoting the ethics of global health research, progress in real life will always be incremental. Sadly, opportunities for documenting and learning from these incremental steps are currently missing.

## Conclusion

Developing a clear, feasible and shared approach to benefit sharing from health research is a long way from a reality in the Kenyan context. In particular, in this study, we have shown that various categories of stakeholders involved in health research in Kenya have a rather limited view of the moral and ethical debates that preceded consideration for benefit sharing and social value as ethical imperatives within global health research.

There is some limited receptivity among stakeholders, to ethical reflection regarding the conduct of health research in these settings. However, this receptivity has not necessarily led to recognition or internalisation of globally discussed principles. Closer attention to the processes through which ethical principles are enacted in practice is needed, including distilling lessons on how best to involve individuals and communities in promoting ethical conduct of global health research in resource poor settings.

## Competing interests

The authors declare that they have no competing interests.

## Authors' contributions

The preparation for and conduct of the study was undertaken by all authors. GML conducted all the interviews, and undertook the analysis supported by MP, RF and EM. All authors contributed to the development of the final manuscript and approve the final version.

## Pre-publication history

The pre-publication history for this paper can be accessed here:

http://www.biomedcentral.com/1472-6939/12/20/prepub
